# Lower incidence of diabetic retinopathy and worsening events after phentermine assisted weight loss across a large U.S. cohort

**DOI:** 10.1038/s41433-025-03818-x

**Published:** 2025-05-10

**Authors:** Madhukar Kumar, Nadia J. Abbass, Matthew W. Russell, Nikhil Das, Rishi P. Singh, Katherine E. Talcott

**Affiliations:** 1 Case Western Reserve University School of Medicine, Cleveland, OH USA; 2 Center for Ophthalmic Bioinformatics, Cole Eye Institute, Cleveland Clinic, Cleveland, OH USA; 3 Cleveland Clinic Lerner College of Medicine of Case Western Reserve University, Cleveland, OH USA; 4 Cleveland Clinic Cole Eye Institute, Cleveland, OH USA; 5 Cleveland Clinic Martin Hospitals, Stuart, FL USA

**Keywords:** Retinal diseases, Risk factors

## Abstract

**Background/Objectives:**

Medication assisted weight loss for treatment of obesity has rapidly increased. The effect of this treatment with phentermine on diabetic retinopathy (DR) is underexplored.

**Subjects/Methods:**

Retrospective cohort study. The U.S. Collaborative Network of the TriNetX platform was queried for patients diagnosed with overweight and obesity and prescription of phentermine. Patients were propensity score matched across baseline demographics and systemic risk factors at the time of medication initiation and compared to those diagnosed with overweight and obesity without usage of weight loss medications, identifying 26,611 patients per cohort after propensity score matching. Risk ratios (RR) of incident DR and related diagnoses or procedural codes found after the medication initiation date for pertinent disease worsening and treatment metrics were assessed.

**Results:**

Phentermine usage was found to be associated with reduced future risk of a new diagnosis of DR with macular oedema (RR 0.462; (95% CI 0.372–0.573), *P* < 0.001), mild non-proliferative diabetic retinopathy (NPDR) (RR 0.621 (0.508, 0.760), *P* < 0.001), moderate NPDR (RR 0.567 (0.402, 0.799), *P* < 0.001), severe NPDR (RR 0.477 (0.233, 0.981), *P* = 0.003), proliferative diabetic retinopathy (PDR) (RR 0.451 (0.323, 0.629), *P* < 0.001), vitreous haemorrhage (RR 0.347 (0.200, 0.602), *P* < 0.001), need for intravitreal anti-VEGF injection (RR: 0.530 (0.354, 0.793), *P* < 0.001).

**Conclusion:**

The present analysis suggests that phentermine usage is associated with a decreased risk of diabetic macular oedema, NPDR, PDR, and worsening events.

## Introduction

Obesity is a growing public health concern, significantly increasing risk of all-cause mortality [1]. While lifestyle interventions such as diet and behavioral modifications are first line in the treatment of obesity, medication assisted weight loss has become an increasingly utilized option for patients who are resistant to conservative medical management [2–4]. This approach involves the utilization of prescription medications in conjunction with a healthy diet and exercise to aide in weight loss and improve overall patient health.

Originally approved by the FDA in 1959 as an appetite-suppressing medication, phentermine was the most commonly prescribed weight loss drug in the United States (US) by several orders of magnitude in the most recent 2015 analysis [5, 6]. Phentermine is a centrally acting amine that primarily increases concentration of norepinephrine, and to a lesser extent dopamine and serotonin, in the synaptic cleft through stimulation of neurotransmitter release and reuptake inhibition [7]. Increased catecholamines in the hypothalamus modulates appetite by reducing the perception of hunger [8]. Additionally, administration of a catecholaminergic agent has been shown to reinforce resting metabolic rate resulting in increased energy expenditure and thus more significant weight loss [9]. This auxiliary mechanism by which phentermine may induce weight loss has only been tested in animal studies to date and has demonstrated mixed results [10, 11]. Prospective clinical trials on phentermine have established robust weight loss with patients experiencing 3.5–7.3% more total body weight loss than placebo [12–16]. Clinical trials of phentermine/topiramate found even greater weight loss at 56 weeks compared to placebo. Patients in the high dose arm lost 10.8% and 9.3%, and patients in the low dose arm lost 8.0% and 3.5% of total body weight in the CONQUER and EQUIP trials respectively [17, 18].

Obesity has been implicated as a driver of diabetic retinopathy (DR). Across a US survey of over 20,000 US diabetic patients, both obesity and BMI were found to be significantly associated with DR after controlling for confounders [19]. Due to these associations, increasing attention has been focused on effects of weight loss and mitigation of DR. A recent meta-analysis examining bariatric surgery patients found significantly reduced post-operative prevalence of DR after surgery compared to non-surgical patients [20]. While these findings are promising, bariatric surgery is invasive and carries long term consequences to patients, making medication assisted weight loss an appealing option for both clinicians and patients. While medication assisted weight loss is efficacious, it has yet to be examined with respect to DR.

## Materials/subjects and methods

The aim of this study is to characterize the effect of medication associated weight loss with phentermine on post-treatment incidence of DR progression, disease worsening events, and pertinent treatment needs using a large platform containing aggregated, standardized, and de-identified medical records data that utilizes International Classification of Diseases (ICD) codes. Data was accessed on February, 2023. The TriNetX U.S. Collaborative Network provides access to electronic medical records information on approximately 120 million patients from 70 contributing healthcare organizations. TriNetX, LLC is a Health Insurance Portability and Accountability Act (HIPAA) compliant, certified to the ISO 27001:2013 standard, and maintains an Information and Security Management System to ensure protection of data.

Using this platform, patients were queried for presence of an RxNorm medication code for phentermine and ICD-10 codes for overweight and obesity and type 1 or 2 diabetes (DM), identifying 26,813 patients. A control cohort of patients with a diagnosis of overweight and obesity along with ICD-10 codes for DR, and no code for phentermine or any other FDA-approved weight loss medication (semaglutide, setmelanoglutide, liraglutide, orlistat, naltrexone, or bupropion) usage was also queried, identifying 2,237,179 patients. Patients with a procedural code for bariatric surgery were excluded in both groups to remove a potential confounding source of systemic improvement. Additionally, both medication and control cohorts excluded patients with a diagnosis of retinal vascular occlusions or degeneration of macular and posterior pole. Any patient who met all inclusion and exclusion criteria within 20 years of the time of data collection in January 2025 were included in the study. Covariate balance among the propensity score matched cohorts was assessed with standard differences where values greater than 0.10 were indicative of imbalance.

The index events were coding for type 1 or 2 DM and overweight and obesity for the control cohort and coding for type 1 or 2 DM, overweight and obesity, and phentermine for the phentermine cohort. Outcomes, which were evaluated between 1 day and 3 years after the index event, included mild non-proliferative diabetic retinopathy (NPDR), moderate NPDR, severe NPDR, proliferative diabetic retinopathy (PDR), vitreous hemorrhage (VH), intravitreal anti-VEGF injection, pan-retinal photocoagulation, pars plana vitrectomy (PPV), and tractional retinal detachment (TRD). To ensure only incident cases were included in the analysis, patients with an ICD-10 code of interest before the index event were excluded from the analysis. All procedural, diagnosis, and prescription codes used in the study design are listed in Supplementary Table 1.

Risk ratios and 95% confidence intervals (CI) were calculated to determine the impact of a phentermine usage on the risk of incident diagnoses of PDR, NPDR, VH, IVI, PRP, PPV, TRD, DME, after the medication trial.

## Results

### Demographics

A total of 26,813 patients were identified to carry a diagnosis of overweight or obesity and either type 1 or 2 diabetes who underwent treatment with phentermine and no other FDA-approved weight loss medication. A total of 2,237,179 patients were identified to carry a diagnosis of overweight or obesity and either type 1 or 2 diabetes who lacked a procedural code for phentermine or any other weight loss medication usage. Propensity score matching was used to match for age, gender, race, ethnicity, BMI, essential hypertension, type of diabetes, long term insulin use, coronary artery disease, hypercholesterolemia, and baseline DR severity. Similarly, baseline hemoglobin A1c (HbA1c) and serum total cholesterol lab values were included in propensity score matching. Before matching, the phentermine cohort had a significantly greater percentage of female and Hispanic patients, younger age, and greater BMI than those who were not treated with phentermine. After matching, both cohorts comprised 26,611 patients and were not significantly different with respect to baseline demographics and systemic disease states, with the exceptions of BMI, and HbA1c (Table 1). Follow-up time was similar between the two cohorts, with a mean of 751 ± 406 days in the phentermine group and a mean of 757 ± 413 days in the control group.

**Table 1:** Demographic information of phentermine and overweight cohorts after Propensity Score Matching.

Variable	Phentermine [SD] or (%) (*N* = 26,611)	Overweight [SD] or (%) (*N* = 26,611)	Standard difference
Age (years)	50.3 ± 13.2	50.3 ± 13.6	0.007
Gender			
Female	18,944 (71.189%)	18,757 (70.486%)	0.016
Male	6876 (25.839%)	7064 (26.545%)	0.016
Race			
White	14,565 (54.733%)	14,589 (54.823%)	0.002
Black	5838 (21.938%)	5796 (21.78%)	0.004
Ethnicity			
Hispanic or Latino	4135 (15.539%)	4109 (15.441%)	0.003
BMI	39.8 ± 8.4	36.3 ± 7.5	0.439
Systemic Diagnoses			
Essential HTN	15,741(59.2%)	15,601 (58.6%)	0.011
Type 1 DM	1464 (5.5%)	1347 (5.1%)	0.020
Type II DM	15,114 (56.8%)	15,071 (56.6%)	0.003
Long term use of insulin	2169 (8.2%)	2053 (7.7%)	0.016
Hypercholesterolemia	4254 (16.0%)	4156 (15.6%)	0.010
CAD	1923 (7.2%)	1893 (7.1%)	0.004
Baseline DR			
Type 1 DM w/ Mild NPDR	33 (0.1%)	10 (0.0%)	0.030
Type 2 DM w/ Mild NPDR	207 (0.8%)	161 (0.6%)	0.021
Type 1 DM w/ Moderate NPDR	10 (0.0%)	10 (0.0%)	<0.001
Type 2 DM w/ Moderate NPDR	50 (0.2%)	37 (0.1%)	0.012
Type 1 DM w/ Severe NPDR	10 (0.0%)	10 (0.0%)	<0.001
Type 2 DM w/ Severe NPDR	29 (0.1%)	12 (0.0%)	0.023
Type 1 DM w/ PDR	23 (0.1%)	10 (0.0%)	0.020
Type 2 DM w/ PDR	78 (0.3%)	46 (0.2%)	0.025
Laboratory Values			
Hemoglobin A1c	6.6 ± 1.7	7.2 ± 2.0	0.305
Serum Cholesterol	178.0 ± 47.8	180.8 ± 49.0	0.058

*SD* standard deviation, *BMI* body mass index, *HTN* hypertension,

*DM* diabetes mellitus, *CAD* coronary artery disease.

### Ocular outcomes

When stratified by category of DR, patients who were managed with phentermine were significantly less likely to experience new onset mild NPDR (RR 0.621, 95% CI 0.508–0.760) moderate NPDR(RR 0.567, 0.402–0.799), severe NPDR (RR 0.479, 0.233–0.981), or proliferative DR (RR 0.451, 0.323–0.629) compared to those who did not undergo treatment (Table 2). Patients who underwent medication assisted weight loss were also significantly less likely to experience complications of diabetic retinopathy worsening such as VH, DME, and IVI (*p* < 0.05 for all, Table 2). Although decreased risk of TRD and needing PRP was observed in phentermine treated patients, this difference was not deemed statistically significant.

**Table 2:** Risk of diabetic retinopathy and complications after phentermine treatment.

Cohorts	Patients in cohort	Patients with outcome	Risk ratio (95% CI)	*P* value
**Mild non-proliferative diabetic retinopathy**				
Phentermine	26,370	152	0.621 (0.508, 0.760)	**<0.001**
Overweight	26,406	245		
**Moderate non-proliferative diabetic retinopathy**				
Phentermine	26,551	51	0.567 (0.402, 0.799)	**0.001**
Overweight	26,559	90		
**Severe non-proliferative diabetic retinopathy**				
Phentermine	26,577	11	0.479 (0.233, 0.981)	**0.040**
Overweight	26,591	23		
**Proliferative diabetic retinopathy**				
Phentermine	26,514	50	0.451 (0.323, 0.629)	**<0.001**
Overweight	26,531	111		
**Diabetic retinopathy with macular edema**				
Phentermine	26,261	119	0.462 (0.372, 0.573)	**<0.001**
Overweight	26,276	258		
**Vitreous hemorrhage**				
Phentermine	26,561	17	0.347 (0.200, 0.602)	**<0.001**
Overweight	26,564	49		
**Intravitreal anti-VEGF injection**				
Phentermine	26,556	36	0.530 (0.354, 0.793)	**0.002**
Overweight	26,563	68		
**Pars plana vitrectomy**				
Phentermine	26,593	11	0.786 (0.357, 1.731)	0.549
Overweight	26,595	14		
**Pan retinal photocoagulation**				
Phentermine	26,584	12	0.546 (0.270, 1.102)	0.086
Overweight	26,590	22		
**Tractional retinal detachment**				
Phentermine	26,598	10	0.588 (0.269, 1.284)	0.178
Overweight	26,589	17		

Supplementary Table 1. Codes utilized in cohort design, propensity score matching, and outcome analysis.

## Discussion

The present study examined associations between phentermine assisted weight loss and DR across a large national platform. Our findings demonstrate that phentermine treatment is associated with decreased risk of new onset DR and DR progression. Patients whose obesity was managed with phentermine were significantly less likely to experience advanced proliferative disease, DME, VH, or require IVI compared to patients who were not treated with phentermine.

The findings of the present study are supported by a meta-analysis of prospective cohort studies that showed obesity to be associated with risk of DR [21]. These results may be largely explained by improvements in systemic health as a result of the medical intervention. Garvey et al. in a post hoc examination of the CONQUER and OB-202/DM-230 trials found phentermine/topiramate usage to be associated with a significant reduction in HbA1c [22]. Lowering HbA1c has also been found to decrease rates of development and progression of DR [23]. Since DR is a microvascular complication of poor systemic diabetic health, it is reasonable to assume that the reduction in proliferative DR incidence and disease worsening events such as VH, or need for intravitreal injection may be explained by overall glycaemic improvement in the phentermine cohort [24]. However, it is important to note that after propensity score matching there was a small, but significant difference in HbA1c and BMI at baseline between phentermine and control cohorts (HbA1c- 6.6 ± 1.7 vs. 7.2 ± 2.0, respectively, BMI- 39.8 ± 8.4 vs. 36.3 ± 7.5, respectively) (Table 1). Future long term, high powered clinical trial data sets examining phentermine and the development of ocular pathology may add credence to the associations presented.

Tight control of hyperglycemia has been well-established to reduce DR and DR worsening [25, 26]. However, intense diabetes control has been reported to worsen DR between 3 months to 3 years [27]. Changes during this critical period via targeted weight loss rather than diabetic therapy was assessed by looking at outcomes from 1 day to 3 years after index event. Strengths of the present study lie in the utilization of a national platform to investigate phentermine administration and its effect on DR and DR worsening. To date, this is the first study to examine these associations. Our results suggest overweight or obese patients treated with medication assisted weight loss may reduce their risk of DR development and progression.

However, the present study is not without its limitations. Despite matching, significant baseline differences in BMI and HbA1C persisted between the cohorts, potentially introducing selection bias. Moreover, while we hypothesized that phentermine-induced weight loss led to improved DR outcomes, there may be an unrecognized pleiotropic effect of phentermine contributing to these results. Additionally, because data obtained through TriNetX is presented in an aggregated form, it is difficult to assess patient-level data such as visual acuity, duration of diabetes diagnosis, lifestyle changes, or specifics of the treatment course such as medication dosage and duration. Finally, as TriNetX relies on ICD-10 and procedural codes to categorize variables, the conclusions drawn from such data is contingent on proper physician coding, leaving room for bias if disease states are coded inconsistently across health care organizations and/or providers.

This study provides novel insight examining medication assisted weight loss and DR across a large national cohort in the US, suggesting a protective association between phentermine and DR. The associations herein indicate a need for further investigation of medication assisted weight loss on development of ocular morbidity and mortality in large long term clinical settings. The results are especially pertinent as medication-assisted weight loss has become more popular with long-term therapies being developed, already-approved medications showing promise in the reduction of BMI, and an increased momentum in prescription and adoption of weight loss medication. Most notably, there are currently six other FDA-approved medications that do not contain phentermine on the market for weight loss with various mechanisms of action: orlistat, naltrexone-bupropion, semaglutide, setmelanotide, and liraglutide, with the most recent being approved in June of 2021 [28]. While their mechanisms of action may differ from that of phentermine, they all provide substantial weight loss. Consequently, these drugs may potentially hold promise in mitigating the development and progression of DR as well as other obesity associated ocular comorbidities.

## Summary

### What was known before:

- Improved metabolic health through bariatric surgery is associated with reduced diabetic retinopathy risk and worsening metrics.

### What this study adds:

- Phentermine as used for medication assisted weight loss is associated with decreased risk of diabetic retinopathy diagnoses, worsening events, and need for medical intervention.

## Supplementary information

Supplemental Table 1.

## Data Availability

The data collected from the TriNetX platform is aggregated and de-identified. As such, it is not possible to share individual data. The aggregate results have been shared in the results of this paper.
