# Supplementary material for: Lower incidence of diabetic retinopathy and worsening events after phentermine assisted weight loss across a large U.S. cohort
Source: Eye (Lond). 2025 May 10;39(11):2183–7. doi: 10.1038/s41433-025-03818-x (PMC12274358; doi:10.1038/s41433-025-03818-x)
Supplement: Supplementary file 1 — Supplemental Table 1. [file 41433_2025_3818_MOESM1_ESM.docx]

**Supplemental Table 1.** Codes utilized in cohort design, propensity score matching, and outcome analysis.

| **Code Type** | **Code** | **Description** |
| --- | --- | --- |
| ICD-10 | H43.1 | Vitreous hemorrhage |
| ICD-10 | H35.3 | Degeneration of macula and posterior pole |
| ICD-10 | H35.3 | Degeneration of macula and posterior pole |
| ICD-10 | H33.4 | Traction detachment of retina |
| ICD-10 | E66 | Overweight and obesity |
| ICD-10 | H34 | Retinal vascular occlusions |
| ICD-10 | E11.351 | Type 2 diabetes mellitus with proliferative diabetic retinopathy with macular edema |
| ICD-10 | E11.35 | Type 2 diabetes mellitus with proliferative diabetic retinopathy |
| ICD-10 | E11.341 | Type 2 diabetes mellitus with severe nonproliferative diabetic retinopathy with macular edema |
| ICD-10 | E11.34 | Type 2 diabetes mellitus with severe nonproliferative diabetic retinopathy |
| ICD-10 | E11.331 | Type 2 diabetes mellitus with moderate nonproliferative diabetic retinopathy with macular edema |
| ICD-10 | E11.33 | Type 2 diabetes mellitus with moderate nonproliferative diabetic retinopathy |
| ICD-10 | E11.321 | Type 2 diabetes mellitus with mild nonproliferative diabetic retinopathy with macular edema |
| ICD-10 | E11.32 | Type 2 diabetes mellitus with mild nonproliferative diabetic retinopathy |
| ICD-10 | E11.311 | Type 2 diabetes mellitus with unspecified diabetic retinopathy with macular edema |
| ICD-10 | E11 | Type 2 diabetes mellitus |
| ICD-10 | E10.351 | Type 1 diabetes mellitus with proliferative diabetic retinopathy with macular edema |
| ICD-10 | E10.35 | Type 1 diabetes mellitus with proliferative diabetic retinopathy |
| ICD-10 | E10.341 | Type 1 diabetes mellitus with severe nonproliferative diabetic retinopathy with macular edema |
| ICD-10 | E10.34 | Type 1 diabetes mellitus with severe nonproliferative diabetic retinopathy |
| ICD-10 | E10.331 | Type 1 diabetes mellitus with moderate nonproliferative diabetic retinopathy with macular edema |
| ICD-10 | E10.33 | Type 1 diabetes mellitus with moderate nonproliferative diabetic retinopathy |
| ICD-10 | E10.321 | Type 1 diabetes mellitus with mild nonproliferative diabetic retinopathy with macular edema |
| ICD-10 | E10.32 | Type 1 diabetes mellitus with mild nonproliferative diabetic retinopathy |
| ICD-10 | E10.311 | Type 1 diabetes mellitus with unspecified diabetic retinopathy with macular edema |
| ICD-10 | E10 | Type 1 diabetes mellitus |
| CPT | 67228 | Treatment of extensive or progressive retinopathy (eg, diabetic retinopathy), photocoagulation |
| CPT | 67028 | Intravitreal injection of a pharmacologic agent (separate procedure) |
| CPT | 43775 | Laparoscopy, surgical, gastric restrictive procedure; longitudinal gastrectomy (ie, sleeve gastrectomy) |
| CPT | 1014238 | Vitrectomy, mechanical, pars plana approach |
| RXNORM | 8152 | Phentermine |
| RXNORM | 7243 | Naltrexone |
| RXNORM | 595060 | Ranibizumab |
| RXNORM | 475968 | Liraglutide |
| RXNORM | 42347 | Bupropion |
| RXNORM | 37925 | Orlistat |
| RXNORM | 253337 | Bevacizumab |
| RXNORM | 2469247 | Setmelanotide |
| RXNORM | 1991302 | Semaglutide |
| RXNORM | 1232150 | Aflibercept |
